# Delayed Development of Coronary Ostial Stenosis following Surgical Aortic Valve Replacement: A Case Report of Unusual Presentation

**DOI:** 10.1155/2018/8512584

**Published:** 2018-04-01

**Authors:** Doosup Shin, Kevin Huang, Igor Sunjic, Michael Berlowitz, Xavier Prida

**Affiliations:** ^1^Department of Internal Medicine, University of South Florida Morsani College of Medicine, Tampa, FL, USA; ^2^Department of Cardiovascular Sciences, University of South Florida Morsani College of Medicine, Tampa, FL, USA

## Abstract

Coronary ostial stenosis is a rare but potentially life-threatening complication that occurs in 1%–5% of patients who undergo surgical aortic valve replacement (SAVR). Symptoms typically appear within the first 6 months and almost always within a year after SAVR. We report an unusually delayed presentation of non-ST segment elevation myocardial infarction due to coronary ostial stenosis 22 months after SAVR. A 71-year-old woman underwent uncomplicated SAVR with a bioprosthetic valve in August 2015 for severe aortic stenosis. A preoperative coronary angiogram demonstrated widely patent left and right coronary arteries. In June 2017, the patient presented to the hospital with chest pain. An electrocardiogram demonstrated 1 mm ST segment depression in the anterolateral leads, and serum troponin I level was elevated to 2.3 ng/ml. Diagnostic coronary angiography revealed severe ostial stenosis (99%) of the right coronary artery. A bare-metal stent was successfully placed with an excellent angiographic result, and the patient was asymptomatic at 4 months of follow-up after the procedure. As seen in our case, coronary ostial stenosis should be considered in the differential diagnosis of chest pain or arrhythmia in patients presenting with a history of SAVR, even if the procedure was performed more than 1 year prior to presentation.

## 1. Introduction

Coronary ostial stenosis is a rare but potentially life-threatening complication associated with surgical aortic valve replacement (SAVR). It has been reported to occur in 1%–5% of all patients who undergo the procedure [1–5]. Coronary ostial stenosis can present with diverse clinical symptoms ranging from angina to acute coronary syndrome (ACS) and even sudden cardiac death [4]. These symptoms typically appear within the first 6 months after SAVR and have rarely been identified beyond 1 year after surgery. We report an unusually delayed clinical presentation that developed 22 months after SAVR.

## 2. Case Presentation

**Figure 1:**
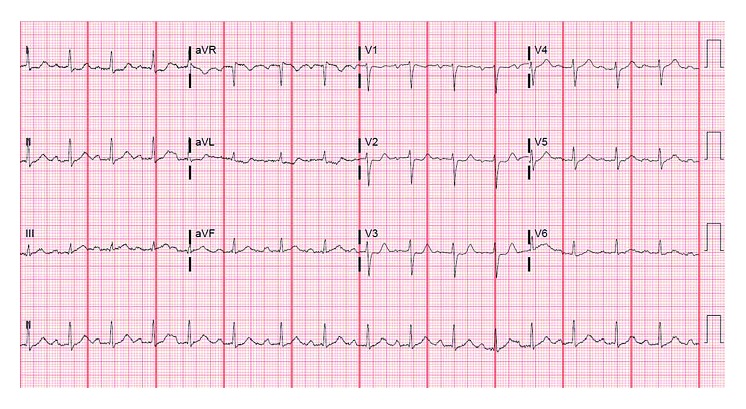
Electrocardiogram on admission demonstrates 1 mm ST segment depression in leads I, aVL, V2, and V3.

**Figure 2:**
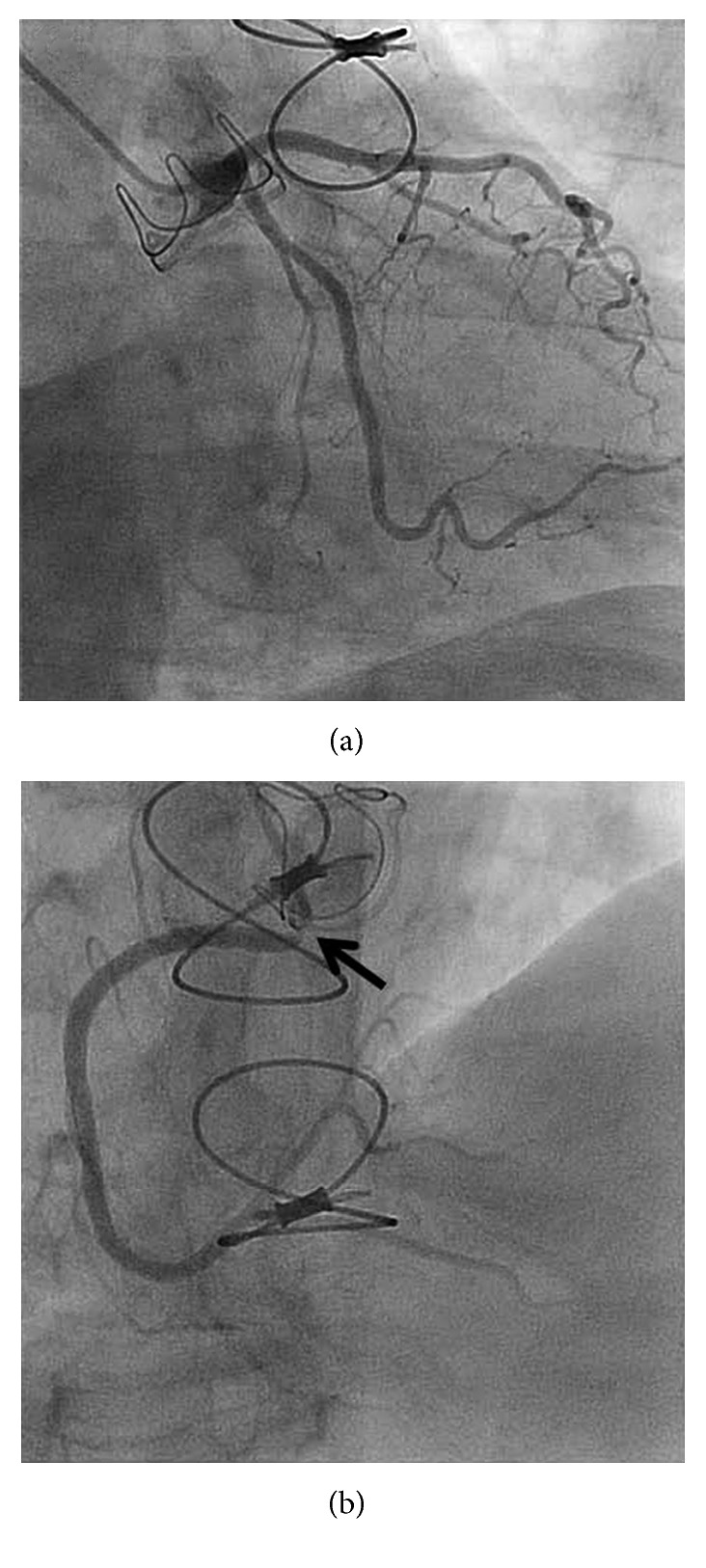
Diagnostic coronary angiogram shows (a) widely patent left coronary arteries and (b) severe ostial stenosis of the right coronary artery (arrow).

**Figure 3:**
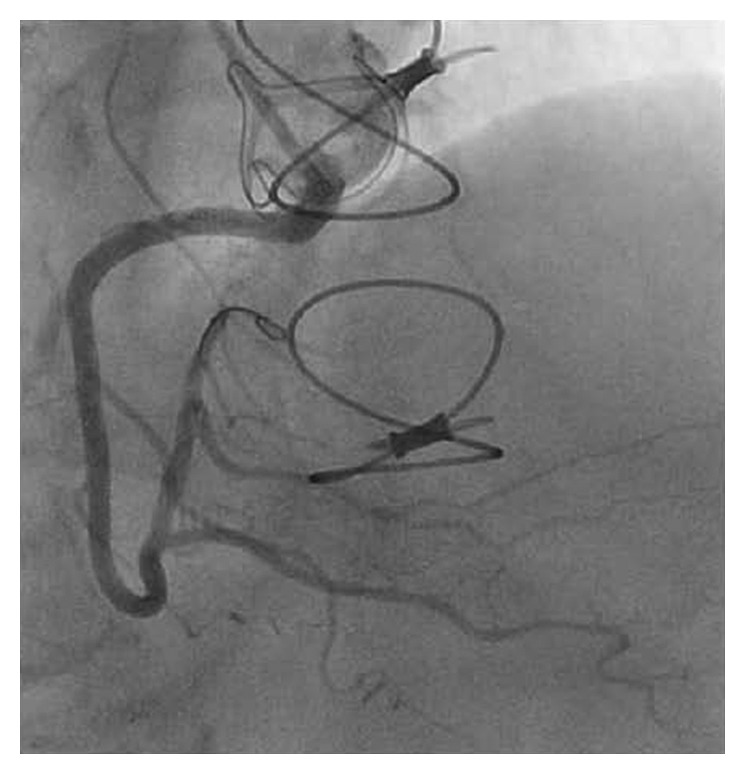
Postprocedure coronary angiogram shows successful revascularization of the right coronary artery with stent placement.

The patient is a 71-year-old woman with a past medical history of hypertension, hyperlipidemia, type 2 diabetes mellitus, and severe aortic stenosis who underwent SAVR in August 2015. A preoperative coronary angiogram revealed normal coronary arteries without evidence of ostial obstruction. SAVR was successfully performed using a 23 mm diameter Magna bioprosthetic valve. During the surgery, antegrade cardioplegia was first administered to achieve electromechanical arrest. From this point, a dose of cold blood retrograde cardioplegia was administered to the coronary sinus every 15 minutes. The patient had an uncomplicated postoperative course and had returned to her usual lifestyle. A postoperative echocardiogram demonstrated a normal ejection fraction and a normally functioning bioprosthetic aortic valve with a mean gradient of 8–10 mmHg across the valve. The patient had been compliant with medications, including aspirin, statin, and beta-blocker. In June 2017, the patient presented with pressure-like exertional chest pain. An electrocardiogram demonstrated 1 mm ST segment depression in leads I, aVL, V2, and V3 (Figure 1). Laboratory investigations revealed elevation of the serum troponin I level to 2.3 ng/ml (normal value: less than 0.032 ng/ml). Guideline-directed medical therapy for non-ST elevation myocardial infarction (NSTEMI) was begun, and the patient was assigned to an invasive strategy. Diagnostic coronary angiography demonstrated isolated severe ostial stenosis (99%) of the right coronary artery with significant catheter pressure waveform dampening but no evidence of thrombus (Figure 2). It showed dramatically tapered appearance from what appeared to be normal arterial segment to a critical ostial stenosis. The lesion was less likely a catheter-induced spasm because (1) there was no direct catheter engagement or deep intubation of the vessel and (2) it persisted despite bolus administration of intracoronary nitroglycerin as well as continuous intravenous infusion of nitroglycerin prior to and during the procedure. Intravascular ultrasound (IVUS) could not be considered due to severity of the stenosis. The lesion was successfully treated using balloon dilations and a single 4.5 mm × 13 mm bare-metal stent (BMS) with excellent angiographic result (Figure 3). The patient was discharged from the hospital the next day without acute complications and was asymptomatic at 4 months of follow-up after the percutaneous coronary intervention (PCI).

## 3. Discussion

In 1967, Roberts and Morrow first described coronary ostial stenosis as a complication of SAVR [6]. It has been reported as a rare but potentially lethal complication following SAVR. Its prevalence has been reported in 1%–5% of patients who undergo SAVR [1–5]. Coronary ostial stenosis secondary to SAVR more commonly affects the left main coronary artery (LMCA) but also affects the right coronary artery [5]. Clinical syndromes include stable angina, ACS, ventricular arrhythmia, congestive heart failure, and sudden cardiac death [4]. These clinical syndromes typically occur within the first 6 months after SAVR. Rarely has coronary ostial stenosis been identified in a patient more than one year after SAVR. To our knowledge, only one study has reported such a case [3]. Our patient represents one of the latest clinical presentations of coronary ostial stenosis occurred 22 months following SAVR.

Several different underlying mechanisms have been suggested to explain the development of this disease entity. In most cases, it has been attributed to the use of ostial cannulation for antegrade cardioplegia during surgery, which can cause a mechanical injury to the coronary ostium resulting in hyperplastic reaction and stenosis [5, 7, 8]. Furthermore, infusion pressure of the cardioplegic solution administered through the cannulation catheter may also contribute to this mechanical injury [9]. However, coronary ostial stenosis can develop after SAVR even without cannulation [10], and brief cannulation times with intermittent infusion of antegrade cardioplegic solution did not eliminate the risk [4, 11]. Similarly, in our case, only a dose of antegrade cardioplegia was given to achieve initial electromechanical arrest, and then, retrograde cardioplegia was used throughout the surgery. Therefore, other mechanisms might also play a role in the development of coronary ostial stenosis after SAVR. First, turbulent flow around the prosthetic valve can cause intimal thickening and fibrous proliferation near the aortic root resulting in ostial stenosis [4–7, 10, 11]. Second, an immunological reaction to the bioprosthetic aortic valve can be another possible mechanism [12, 13]. One study supported this immunological hypothesis by using IVUS which demonstrated a massive extravascular fibrosis and its extrinsic compression around the ostium of the coronary artery causing severe ostial stenosis after SAVR [13].

Revascularization alternatives for ostial coronary artery stenosis following SAVR include coronary artery bypass graft and PCI. Availability/selection of appropriate management strategies for ostial coronary artery stenosis, particularly unprotected LMCA disease, remains a controversial subject. With technological advancement, PCI and stent placement can successfully treat coronary ostial stenosis with good early and late outcomes [5, 7, 12, 14–17]. This approach was applied in our patient with good short-term outcomes. We chose the BMS over the drug-eluting stent (DES) due to its good radial strength and tightness of the lesion in the setting of a large vessel diameter. This anatomic consideration should be balanced with the risk of restenosis, especially in the aortocoronary ostial lesions. At the same time, the patient's characteristics and the risk of bleeding should also be considered when selecting the type of stent, as DES placement would require longer dual-antiplatelet therapy [16].

In our case, further characterization of the ostial lesion by using prestenting IVUS was not possible because the IVUS catheter could not be negotiated through the critical narrowing of the lesion. Furthermore, the length of the procedure and the patient's disquiet did not allow us to extend the procedure for poststenting IVUS. Although IVUS could not be done in our case, it should be considered in future cases to better understand possible underlying mechanism and optimize stenting.

## 4. Conclusion

Although coronary ostial stenosis typically occurs within the first 6 months after SAVR, we describe its identification in association with NSTEMI 22 months after the surgery. Therefore, coronary ostial stenosis should be considered in the differential diagnosis of chest pain or arrhythmia in patients presenting with a history of SAVR, even if the procedure was performed more than 1 year prior to presentation. Also, we should continue to have a higher index of suspicion in these patients for ACS even after sufficient time has passed.

## References

[1] Yates J. D., Kirsh M. M., Sodeman T. M., Walton J. A., Brymer J. F. (1974). Coronary ostial stenosis: a complication of aortic valve replacement. *Circulation*.

[2] Sethi G. K., Scott S. M., Takaro T. (1979). Iatrogenic coronary artery stenosis following aortic valve replacement. *Journal of Thoracic and Cardiovascular Surgery*.

[3] Pennington D. G., Dincer B., Bashiti H. (1982). Coronary artery stenosis following aortic valve replacement and intermittent intracoronary cardioplegia. *The Annals of Thoracic Surgery*.

[4] Pillai J. B., Pillay T. M., Ahmad J. (2004). Coronary ostial stenosis after aortic valve replacement, revisited. *The Annals of Thoracic Surgery*.

[5] Ziakas A. G., Economou F. I., Charokopos N. A. (2010). Coronary ostial stenosis after aortic valve replacement: successful treatment of 2 patients with drug-eluting stents. *Texas Heart Institute Journal*.

[6] Roberts W. C., Morrow A. G. (1967). Late postoperative pathological findings after cardiac valve replacement. *Circulation*.

[7] Funada A., Mizuno S., Ohsato K. (2006). Three cases of iatrogenic coronary ostial stenosis after aortic valve replacement. *Circulation Journal*.

[8] Trimble A. S., Bigelow W. G., Wigle E. D., Silver M. D. (1969). Coronary ostial stenosis: a late complication of coronary perfusion in open-heart surgery. *Journal of Thoracic and Cardiovascular Surgery*.

[9] Turillazzi E., Di Giammarco G., Neri M., Bello S., Riezzo I., Fineschi V. (2011). Coronary ostia obstruction after replacement of aortic valve prosthesis. *Diagnostic Pathology*.

[10] Rath S., Goor D. A., Har-Zahav Y., Buttler A., Ziskind Z. (1988). Coronary ostial stenosis after aortic valve replacement without coronary cannulation. *American Journal of Cardiology*.

[11] Force T. L., Raabe D. S., Coffin L. H., DeMeules J. D. (1980). Coronary ostial stenosis following aortic valve replacement without continuous coronary perfusion. *Journal of Thoracic and Cardiovascular Surgery*.

[12] Tsukiji M., Akasaka T., Wada N. (2004). Bilateral coronary ostial stenosis after aortic valve replacement with freestyle stentless bioprosthesis: a case report. *Journal of Cardiology*.

[13] Han S. W., Kim H. J., Kim S., Ryu K. H. (2009). Coronary ostial stenosis after aortic valvuloplasty (comprehensive aortic root and valve repair). *European Journal of Cardio-Thoracic Surgery*.

[14] Khan M. A., Prati F., El-Omar M. (2013). Iatrogenic coronary ostial stenosis of left main stem following aortic valve replacement: visualization with optical coherence tomography. *Cardiovascular Revascularization Medicine*.

[15] Marti V., Auge J. M., Garcia Picart J., Guiteras P., Ballester M., Obrador D. (1995). Percutaneous transluminal coronary angioplasty as alternative treatment to coronary artery bypass surgery in iatrogenic stenosis of the left main coronary artery. *Journal of Interventional Cardiology*.

[16] Bernelli C., Bezante G. P., Brunelli C., Balbi M. (2012). Iatrogenic left main coronary ostial stenosis after a Bentall procedure in an asymptomatic young man. *Texas Heart Institute Journal*.

[17] Cola C., Yuste V. M., Sabate M. (2009). Left main coronary artery stenosis following surgical valve replacement: changing valvular into ischemic heart disease. *Journal of Invasive Cardiology*.

